# Monoclonal antibodies against all known variants of EspA: development of a simple diagnostic test for enteropathogenic *Escherichia coli* based on a key virulence factor

**DOI:** 10.1099/jmm.0.076323-0

**Published:** 2014-12

**Authors:** Uta Praekelt, Rolf Reissbrodt, Andreas Kresse, Asalapuram Pavankumar, Krishnan Sankaran, Roger James, Mary Jesudason, Shalini Anandan, Agila Prakasam, Veeraraghavan Balaji, Shanta Dutta, Sanjucta Dutta, Thandavarayan Ramamurthy, Renate Fischer, Peter Sander, Reiner Schaumann, Armando Navarro, Peter Williams

**Affiliations:** 1 Department of Genetics, University of Leicester, Leicester, UK; 2 Abteilung für Infektionskrankheiten, Robert Koch Institut, Wernigerode, Germany; 3 Centre for Biotechnology, Anna University, Chennai, India; 4 Department of Infection, Immunity and Inflammation, University of Leicester, Leicester, UK; 5 Christian Medical College, Vellore, India; 6 National Institute of Cholera and Enteric Diseases, Kolkata, India; 7 R-Biopharm AG, Darmstadt, Germany; 8 Institut für Medizinische Mikrobiologie, Universität Leipzig, Leipzig, Germany; 9 Department of Public Health, Faculty of Medicine, National Autonomous University of Mexico, Mexico City, Mexico

## Abstract

Enteropathogenic *Escherichia coli* (EPEC) are a major cause of infant diarrhoea in developing countries and a significant public health issue in industrialized countries. Currently there are no simple tests available for the diagnosis of EPEC. Serology of O-antigens is widely used routinely in many laboratories throughout the world, even though it has been known for many years to be an unreliable indicator of EPEC virulence. We have developed a simple, low-cost immunodiagnostic test based on the EspA filament, an essential virulence factor of EPEC and the related enterohaemorrhagic *E. coli* (EHEC). Using recombinant proteins of the five major variants of EspA as immunogens, we raised a panel of three monoclonal antibodies in mice that detects all variants of the native target in bacterial cultures. The antibodies proved suitable for application in sandwich-type assays, including ELISA and lateral flow immunoassays (LFI). Prototypes for both assays were specific for EPEC and EHEC strains when tested against a panel of control micro-organisms. We have also developed a simple, affordable culture medium, A/E medium, which optimizes expression of EspA allowing improved sensitivity of detection compared with standard Dulbecco’s modified Eagle’s medium. Together these reagents form the basis of robust, informative tests for EPEC for use especially in developing countries but also for routine screening in any clinical laboratory.

## Introduction

Enteropathogenic *Escherichia coli* (EPEC) are a major cause of infant diarrhoea in developing countries, accounting for an estimated 10 % of the approximately 1.4 billion paediatric diarrhoeal episodes annually in children under the age of 5 (O’Ryan *et al.*, 2005). In the absence of treatment, particularly among very young children also affected by malnutrition, EPEC diarrhoea can be fatal or lead to irreversible damage to the intestine (Chen & Frankel, 2005). Industrialized countries have experienced an overall decline in childhood diarrhoea during the past 50 years, but EPEC still accounts for a similar proportion of diarrhoeal incidences (Afset *et al.*, 2003; Robins-Browne *et al.*, 2004). In addition in industrialized countries, the closely related pathotype enterohaemorrhagic *E. coli* (EHEC) is responsible for occasional, mainly food-borne outbreaks of diarrhoea in adults and children, frequently accompanied by severe complications such as haemorrhagic colitis and haemolytic uraemic syndrome due to the action of shigatoxins not present in EPEC (Frankel *et al.*, 1998; Smith *et al.*, 2004).

EPEC and EHEC are a heterogeneous group of *E. coli* strains. For many years the diagnosis of EPEC has been based primarily on the identification of O : H serotypes according to WHO guidelines dating from 1987, which recognized the 12 so-called classical EPEC serogroups associated with childhood diarrhoea: O26, O55, O86, O111, O114, O119, O125, O126, O127, O128, O142 and O158 (Campos *et al.*, 2004). EHEC strains are commonly associated with serogroups O103, O145 and O157, while some serogroups, in particular O55, O26 and O111, include both EPEC and EHEC strains. However, the serotyping scheme was developed before EPEC and EHEC virulence mechanisms were elucidated and many subsequent studies have shown that there is only partial correlation between serology and pathotype (Afset *et al.*, 2003; Ajjampur *et al.*, 2008; Campos *et al.*, 2004; Yang *et al.*, 2007). Nevertheless, despite the fact that O-serology is non-informative of virulence, it continues to be used in many clinical laboratories throughout the world as one of the routine tests to establish the cause of diarrhoea (Kozub-Witkowski *et al.*, 2008). While EHEC can be identified by immunological tests for shigatoxins, there is a real need for simple diagnostic tests for EPEC, based on known virulence factors, especially in developing countries where EPEC diarrhoea is endemic, but also in industrialized countries where studies indicate that EPEC may be more prevalent than was previously thought.

EPEC and EHEC colonize the intestinal epithelium causing attaching and effacing (A/E) lesions by a mechanism that involves the intimate attachment of bacteria to the host cell (Kenny *et al.*, 1997). Various virulence factors essential for this process are encoded on a pathogenicity island, the locus of enterocyte effacement, including intimin, a bacterial membrane adhesion protein encoded by the *eae* gene and the EspA (*E. coli* secreted protein A) filament, a hollow tube that acts like a molecular syringe for delivery of the Tir (translocated intimin receptor) protein and other effector molecules into the host cell (Crepin *et al.*, 2005; Frankel *et al.*, 1998; Knutton *et al.*, 1998). Methods that target the presence of virulence genes, such as PCR and DNA microarray tests for the *eae* gene, are ideal as the basis for reliable diagnostic tests, but such methods are generally not applicable to routine diagnostic testing in peripheral health centres in developing countries where resources and skills may be limited. In these circumstances, simple antibody-based tests are much more suitable.

There have been several reports of antibodies raised against various secreted or surface-located EPEC virulence factors (Batchelor *et al.*, 1999; Girón *et al.*, 1995; Kühne *et al.*, 2004; Lu *et al.*, 2002; Menezes *et al.*, 2010); however, these either detected only a limited subset of EPEC strains or required denaturation of the target for detection and to our knowledge have not been developed further. We chose the EspA filament, a 5-stranded helical polymer of identical 21 kDa monomers (Daniell *et al.*, 2003; Delahay *et al.*, 1999), as the immunological target for the development of a low cost EPEC diagnostic test initially intended for use primarily in the Indian subcontinent. We characterized *espA* gene sequences from a set of clinical isolates collected in south India and identified five major variants, all of which were represented, sometimes with minor variations, in the DNA and protein databases. Using recombinant proteins of these five variants as immunogens, we raised monoclonal antibodies capable of detecting all the EspA variants published to date. We also designed a low cost medium for optimal expression of EspA in culture. Together these reagents comprise a simple and reliable replacement for O-serogrouping for the identification of EPEC diarrhoea.

## Methods

### 

#### Bacterial strains and growth conditions.

Clinical isolates were obtained from the following laboratories: 16 *eae^+^* strains from Christian Medical College (CMC), Vellore, India and four *eae^+^* strains from the Centre for Biotechnology (CBT), Anna University, Chennai, India of known O : H serotype; 61 strains from the National Institute of Cholera and Enteric Diseases (NICED), Kolkata, India, isolated on the basis of a positive PCR for intimin (*eae^+^*) but of unknown serotype; 242 strains from the Institut für Medizinische Mikrobiologie, Universität Leipzig, Germany, isolated on the basis of O-serogroups typical for EPEC and EHEC, and of these only 104 were *eae^+^*; 34 *eae^+^* strains from the Department of Public Health, Faculty of Medicine, National Autonomous University of Mexico with known O : H serotypes; 14 *eae^+^* strains from the Robert Koch Institut (RKI), Wernigerode, Germany, which had been O : H-serotyped and also tested for virulence factors associated with EHEC to distinguish EPEC (8 strains) from EHEC (3 *stx1* and 3 *stx2* strains). Non-EPEC reference strains (as listed in Fig. 6) were also from RKI.

Strains were maintained on Luria agar. To induce production of virulence factors, strains were inoculated into Dulbecco’s modified Eagle’s medium (DMEM; Gibco) containing 1 % glucose and incubated overnight at 37 °C in 5 ml volumes without agitation.

#### Improved medium for virulence induction.

To improve the expression of EspA in some strains, we developed an alternative medium, A/E medium, based on soy peptone and yeast extract, using some poorly expressing strains as indicators of improvement in a dot blot (see below). Taking into account the reported importance of calcium and sodium bicarbonate (Abe *et al.*, 2002), and considering the components of DMEM that might be important, such as vitamins, we arrived at the following formulation: 4.5 g Difco Select Soytone l^−1^ (BD); 6 g HEPES l^−1^ (free acid; Melford); 2 g yeast extract l^−1^ (Oxoid); 10 g lactose l^−1^ (Fisher Scientific); 0.2 g CaCl_2_ . H_2_O l^−1^ (Sigma); 0.2 g ferric ammonium chloride l^−1^ (Sigma); and 0.4 g KCl l^−1^ (Sigma). The medium was sterilized either by autoclaving or by filter-sterilization. NaHCO_3_ powder (Sigma) was added to a final concentration of 7.5 g l^−1^ immediately prior to use. Cultures (5–10 ml) were inoculated and incubated overnight at 37 °C without agitation.

#### Cloning and mutagenesis.

Variant *espA* genes were amplified by PCR from genomic DNA using flanking primers UP1 F/UP1 R (727 bp) or UP2 F/UP2 R (1010 bp; Table 1). Products were purified from agarose gels and the DNA sequences determined using the same primers. For cloning and expression of recombinant proteins, *espA* coding regions were amplified from five strains using primers EspA F1 and EspA R1 (isolate III-3, EspA α; 591 bp); EspA F2 and EspA R2 (isolate A5, EspA β and isolate A7, EspA δ; 592 bp); EspA F5 and EspA R2 (isolate III5, EspA γ; 592 bp) and EspA F6 and EspA R4 (isolate C2; EspA ϵ; 582 bp). PCR products were digested with the appropriate restriction enzymes (as indicated in Table 1) and cloned into similarly cut vector pET28a (Novagen) in *E. coli* strain XL10 Gold (Stratagene). After confirmation by DNA sequencing using primer T7 (homologous to vector sequence) the recombinant plasmids were transformed into strain BL21(DE3) (Stratagene).

**Table 1.** Primers for cloning and mutagenesis

Primer	Sequence (5′ to 3′)	*T*_m_ (°C)
UP1 F	TAATACATTATTAATGATTGGTAAAG	54.8
UP1 R	TATCGYTATTTACRTTAAGCATAG	56.6
UP2 F	CTCGGGTATCGATTGTCGAAGAT	67.4
UP2 R	CAGAGGGCGTCACTAATGAGTG	66.2
EspA F1 (α; *Nde*I)	GC*CATATG*GATACATCAACTACAGCAC	67.2
EspA R1 (α; *Sac*I)	GC*GAGTCT*TATTTACCAAGGGATATTCCTG	70.0
EspA F2 (β,δ; *Nde*I)	GC*CATATG*GATACATCAACTGCAACATC	70.4
EspA R2 (β,δ,γ; *Sac*I)	GC*GAGCTC*TTATTTACCAAGGGATATTGCTG	72.7
EspA F6 (ϵ; *Nco*I)	CA*CCATGG*ATAATTCAGTTACATCATC	65.2
EspA R4 (ϵ; *Xho*I)	CG*CTCGAG*TTTACCAAAACTTATTGCC	70.4
EspA F5 (γ; *Nde*I)	GC*CATATG*GATACATCAAATGCAACATC	70.1
Epi F21 (β,δ; *Bam*HI)	GC*GGATCC*ATGAAAGCCAAACTTCCTCAA	77.6
Epi R12 (β,δ; *Hin*dIII)	GC*AAGCTT*ATTTACCAAGGGATATTG	65.4
Epi F20 (β; *Bam*HI)	GC*GGATCC*ATGGATACATCAACTGCAACA	77.2
Epi R1 (β; *Hin*dIII)	GC*AAGCTT*AAATGTCATTGCGAGGATCATT	73.5
Epi F12 (δ; *Bam*HI)	GC*GGATCC*ATGGATACATCAACTGCAACA	77.2
Epi R10 (δ; *Hin*dIII)	GC*AAGCTT*ACGTTTGCAAATCACCAGCGCC	80.1
Epi F6 (ϵ; *Bam*HI)	GC*GGATCC*ATGGATAATTCAGTTACATCA	72.1
Epi R9 (ϵ; *Hin*dIII)	GC*AAGCTT*AGGTTTGCAGGTCACCAGCACC	78.7
Epi F7 (ϵ; *Bam*HI)	GC*GGATCC*ATGGCCAATTTGGTGGATGCC	83.3
Epi R5 (ϵ; *Hin*dIII)	GC*AAGCTT*ATTTACCAAAACTTATTGC	64.7
Epi R20 (ϵ; *Hin*dIII)	GC*AAGCTT*AAAGCTGGTTATTATTCACCGT	70.3
Mut 1 (β N112K)	GTGATTGACTATATAAAAGATCCTCGCAATG	67.7
Mut 2 (β N112K)	CATTGCGAGGATCTTTTATATAGTCAATCAC	67.7
Mut 15 (β D117G)	CTATATAAATGATCCTCGCAATGGCATTACA…	76.8
…GTAAGTGGTATTAGCG
Mut 16 (β D117G)	CGCTAATACCACTTACTGTAATGCCATTGCG…	76.8
…AGGATCATTTATATAG
Mut 5 (δ N96E)	GTTCAGAGCAGTTCTGAGAAGGATGCGAAAGCCAAACTT	80.2
Mut 6 (δ N96E)	AAGTTTGGCTTTCGCATCCTTCTCAGAACTGCTCTGAAC	80.2
Mut 7 (δ K112Q)	GTGATCGACTATATACAAGATCCTCGTAATGGCATTAC	72.2
Mut 8 (δ K112Q)	GTAATGCCATTACGAGGATCTTGTATATAGTCGATCAC	72.2
Mut 17 (ϵ E94D)	CTGAAGTTCAGTCTAGTTCGGACAAGGATAAA…	77.0
…AAGGTCAAACTTC
Mut 18 (ϵ E94D)	GAAGTTTGACCTTTTTATCCTTGTCCGAACTA…	77.0
…GACTGAACTTCAG
T7	AATACGACTCACTATAGGG	51.2

Where appropriate, primer names indicate EspA type and restriction sites (in italics) used for cloning into vector DNA. Primers used for mutagenesis (Mut) also indicate the amino acid substitution achieved (underlined). *T*_m_ values are based on the *T*_m_ Calculator from Thermo Scientific.

For epitope mapping, overlapping fragments of the *espA* genes from the three variants representing the three mAbs used in this work were cloned into pET28a. Templates for amplification were recombinant plasmids containing EspA β for mapping mAb 14, EspA δ for mapping mAb 209 and EspA ϵ for mapping mAb 2. EspA β fragments were amplified using primer combinations Epi F21/Epi R12 (299 bp; amino acids 110–192) and Epi F20/Epi R1 (371 bp; amino acids 1–118). EspA δ fragments were amplified with primers Epi F12/Epi R10 (428 bp; amino acids 1–137) and Epi F21/Epi R12 (299 bp; amino acids 100–192). EspA ϵ fragments were amplified with primers Epi F6/Epi R9 (422 bp; amino acids 1–135), Epi F7/Epi R5 (356 bp; amino acids 78–190) and Epi F6/Epi R20 (485 bp; amino acids 1–156). Fragments were digested with restriction enzymes as indicated in Table 1 and cloned into similarly digested pET28a.

EspA mutant proteins containing single amino acid changes were produced by first amplifying two separate PCR fragments primed with overlapping internal primers containing the appropriate base changes and external primers, and then mixing the resulting fragments together and amplifying with external primers only. The resulting products were digested with restriction enzymes, cloned and expressed in pET28a using the following combinations: EspA β (N112K) Mut 1/EspA R2 (268 bp) and Mut 2/EspA F2 (354 bp); EspA β (D117G) Mut 15/EspA R2 (261 bp) and Mut 16/EspA F2 (378 bp); EspA δ (N96E) Mut 5/EspA R2 (317 bp) and Mut 6/EspA F2 (313 bp); EspAδ (K112Q) Mut 7/EspA R2 (269 bp) and Mut 8/EspA F2 (361 bp); EspA ϵ (E94D) Mut 17/EspA R4 (319 bp) and Mut 18/EspA F6 308 bp).

#### Recombinant protein expression and purification.

Recombinant plasmids were transformed into BL21(D3) (Stratagene) by the calcium chloride method. Transformants were grown at 37 °C to mid-exponential phase in 400 ml Luria broth, induced by addition of IPTG to 1 mM and grown for a further 4 h. Recombinant proteins were isolated in the form of inclusion bodies. Cells in 50 ml culture volumes were pelleted by centrifugation and resuspended in 2.5 ml 0.1 M sodium phosphate (pH 8.5), 0.3 M sodium chloride. After incubation for 30 min at 37 °C in the presence of lysozyme (10 mg ml^−1^) the cells were disrupted by sonication on ice. Suspensions were centrifuged at 6000 r.p.m. for 10 min in an Eppendorf Minispin centrifuge and pellets were resuspended in wash buffer (50 mM Tris, pH 8, 0.1 M sodium chloride, 2 M urea, 0.5 % Nonidet) by syringing to break up clumps. Washing was repeated and suspensions were stored in wash buffer at −20 °C overnight, resulting in almost complete clearing of the suspension. Suspensions were centrifuged at 13 500 r.p.m. for 5 min and the clear supernatants were removed and dialysed against several changes of PBS. Essentially pure EspA protein, which was recovered as a fine translucent precipitate, was quantified by SDS-PAGE against dilutions of a BSA standard.

#### mAb production.

The five major *espA* variants (as shown in Fig. 2) were used as targets for raising monoclonal antibodies. BALB/c mice were immunized with recombinant EspA proteins from different EPEC strains, presented in the form of inclusion bodies, either individually or as mixtures, in the presence of Freund’s adjuvant or Titermax and boosted on two or three subsequent occasions at two-weekly intervals. Serum titres were determined by whole cell ELISA (see below). Splenocytes from mice with a high antibody titre were fused with NS0 cells by standard procedures using PEG and dispensed into 96-well plates. Hybridomas were again screened by whole cell ELISA and positive wells were cloned twice and expanded for production of antibody and storage of cell lines. Large-scale production and purification of antibody were carried out by Sifin Diagnostics and supplied at 1–2 mg ml^−1^. Antibodies were isotyped using a mouse monoclonal antibody isotyping kit (Zymed Laboratories). Microtitre plates (NUNC Maxisorp) were coated with each antibody at 10 µg ml^−1^ in coating buffer (3.03 g Na_2_CO_3_ l^−1^, 6.0 g NaHCO_3_ l^−1^ in distilled water, pH 9.6). ELISA was performed according to the manufacturer’s instructions.

#### Western blots and dot blots.

Proteins were resolved on 12 % polyacrylamide gels and blotted onto nitrocellulose membranes. These were blocked in PBS containing 4 % skimmed milk and probed with hybridoma supernatants at dilutions of 1 : 100 to 1 : 500 for 1 h. Bound antibody was detected with goat antimouse horseradish peroxidase (Sigma) for 30 min, followed by tetramethyl benzidine (TMB) for up to 3 min. Membranes were washed with distilled water and dried, and the results were recorded by scanning. For dot blots, 2 µl volumes of culture were mixed with an equal volume of ethanol, spotted onto polyvinylidene fluoride membrane and dried before processing as for Western blots.

#### Direct whole cell ELISA.

EPEC strains expressing EspA variants were grown overnight in DMEM containing 1 % glucose. Cultures were either mixed together in equal proportions (for initial screening of hybridomas) or used separately (for subsequent testing) and diluted with an equal volume of coating buffer. For testing, 100 µl of mixture was dispensed into each well of 96-well microtitre plates (NUNC Maxisorp), dried overnight at 37 °C, washed twice with PBS and then blocked for 30 min with PBS containing 4 % skimmed milk. Doubling dilutions of serum from test bleeds or undiluted hybridoma supernatants were added to wells and incubated for 1 h, washed three times with PBS, incubated with goat antimouse alkaline phosphatase (Sigma) for 30 min and then washed five times with PBS. Detection was by *p*-nitrophenyl phosphate (Sigma); reactions were stopped by the addition of 1/4 volume of 3 M sodium hydroxide and absorbance was read at 405 nm.

#### Sandwich ELISA.

Purified mAbs were conjugated to horseradish peroxidase using the Lightning-Link kit (Innova Biosciences) according to the manufacturer’s instructions. Microtitre plates were coated with purified mAbs, either individually or as a mixture, at 10 µg ml^−1^ in coating buffer and stored at 4 °C overnight. After rinsing twice with PBS the plates were blocked with 4 % skimmed milk in PBS for 30 min. Overnight cultures were added to wells either directly or after dilution with PBS and incubated for 1 h. After five washes detection was by horseradish peroxidase-conjugated mAbs, again either separately or as a mixture, followed by substrate TMB (liquid substrate system for ELISA, Sigma). After 15 min results were recorded by scanning.

#### R-Biopharm prototype sandwich ELISA.

The sandwich ELISA was carried out in 96-well plates with 12 separate strips of eight wells each in a strip holder, which were supplied ready coated with a mixture of mAbs, stabilized and blocked. All solutions except wash buffer and sample diluent were provided in dropper bottles. To carry out the test, samples were diluted with an equal volume of diluent (protein-buffered NaCl solution) and 100 µl was applied to each well, followed immediately by two drops of conjugate 1 (biotin-conjugated mAbs in stabilized protein solution). After incubation for 1 h at room temperature, wells were rinsed five times with wash buffer (PBS containing 0.1 % thimerosal) and incubated for 30 min with two drops of conjugate 2 (streptavidin-enhanced horseradish peroxidase conjugate in stabilized protein solution). After five washes two drops of substrate (hydrogen/TMB ready to use) were added and left to incubate for 15 min. Results were recorded either by scanning for a visual record, or by stopping the reaction with one drop of stop solution (2 M H_2_SO_4_) followed by measurement of absorbance at 450 nm.

#### Lateral flow immunoassay (LFI).

A prototype LFI was developed in collaboration with Forsite Diagnostics Ltd, York, UK. Purified mAbs were adsorbed to blue latex beads and incorporated into the release pad. The capture antibody was line sprayed onto Prima 40 nitrocellulose membranes (GE Health Care), initially using a single antibody for each device. A control line to indicate a functional assay contained antimouse antibody for capture of the mAb-conjugate. Devices were assembled into cassettes and supplied in sealed foil pouches. For the final prototype device all three antibodies were applied as mixtures to both release pad and capture line. For the assay, bacterial cultures were grown overnight in A/E medium, mixed with 1/10 volume of 5 M sodium chloride and applied either to the devices directly or following brief centrifugation to remove bacterial cells; 70 µl of suspensions or supernatants were applied to the test devices and results recorded photographically after 5 min.

## Results

### EspA sequences of strains from south India

**Fig. 1.**
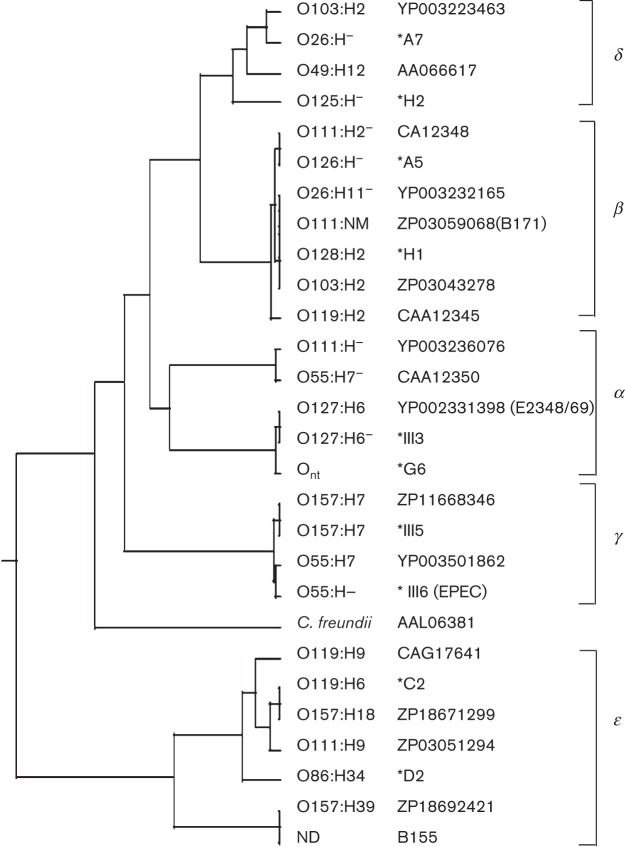
Phylogenetic tree of deduced EspA amino acid sequences using clustal w. *Representative clinical isolates from south India for which *espA* gene sequences were determined in this study (Table 2). B155 represents a strain of unknown serotype from NICED. All others are sequences from the DNA (GenBank) and protein (NCBI) databases. NM, non-motile; O_nt_, non-typable for O-antigen; nd, serotype not determined.

The EspA proteins encoded by the 21 confirmed EPEC or EHEC strains from south India (Table 2) were found to be either identical or closely related to published EspA protein sequences. A phylogenetic tree illustrating the relationship of these EspA proteins to those in protein and DNA databases is shown in Fig. 1, which also includes the EspA sequences of B155, an A/E lesion-forming strain of unknown serotype from NICED (see below) and *Citrobacter freundii*, an organism that is commonly found in soil and water but may be present in the intestinal tract of humans and animals (Tschäpe *et al.*, 1995).

**Table 2.** Clinical isolates from India

Leic. no.	Collection	Serotype	EspA type	GenBank
III 3*	RKI 05-02944	O127 : H6	α	KJ549668
B5	RKI 04-02142	O127 : H6	α	KJ549669
E4	RKI 04-02177	O127 : H6	α	KJ549670
F6	RKI 04-02191	O127 : H6	α	KJ549671
G6*	RKI 04-02203	O_nt_ : H^−^	α	KJ549672
B3	RKI 04-02140	O_nt_ : H^−^	β	KJ549673
H1*	RKI 04-02210	O128 : H2	β	KJ549674
G1	RKI 04-02198	O128 : H2	β	KJ549675
A5*	RKI 04-02130	O126 : H^−^	β	KJ549676
H3	RKI 04-02212	O126 : H^−^	β	KJ549677
III 5*	RKI 05-02946	O157 : H7	γ	KJ549678
III 14	RKI 05-02948	O157 : H7	γ	KJ549679
III 6*	RKI 05-02947	O55 : H^−^	γ	KJ549680
H2*	RKI 04-02211	O125 : H^−^	δ	KJ549681
A6	RKI 04-02131	O125ab : H^−^	δ	KJ549682
A7*	RKI 04-02132	O26 : H^−^	δ	KJ549683
C2*	RKI 04-02151	O119 : H6	ϵ	KJ549684
G2	RKI 04-02199	O119 : H6	ϵ	KJ549685
D2*	RKI 04-02163	O86 : H34	ϵ	KJ549686
F5	RKI 04-02190	O55 : H^−^	ϵ	KJ549687
H6	RKI 04-02215	O55 : H^−^	ϵ	KJ549688
B155*	NICED B155	nd	ϵ	KJ549689

Strains were collected at CBT and CMC; B155 was from NICED. All except B155 were logged and serotyped at RKI and *espA* DNA sequences were determined at the University of Leicester. Deduced EspA protein sequences were classified into five subtypes according to similarity and denoted by α, β, γ, δ or ϵ.

* Strains included in the phylogenetic tree shown in Fig. 1; O_nt_ non-typable for O-antigens of classical EPEC/EHEC strains (O26, O55, O86, O103, O111, O114, O119, O125, O127, O128, O126, O142 O145, O157); nd, not determined.

To rationalize the nomenclature of EspA we have assigned the sequences to five groups based on similarity and designated them using letters of the Greek alphabet by analogy with the nomenclature for intimin. Three of these groups, EspA α, EspA β and EspA γ, exemplified by the well-characterized serotypes O127 : H6, O111 : H2 and O157 : H7, respectively, had already been designated as such in accordance with the intimin variants carried by these strains. We suggest the designation EspA δ for the group of variants including O49 : H12, which was previously named EspA ‘beta variant’ (Bertin *et al.*, 2004), and EspA ϵ for the group including O119 : H6 that showed the greatest sequence divergence of all variants and which had been variously called EspA ‘beta variant’ or EspA ‘β2’ (Afset *et al.*, 2006; Garrido *et al.*, 2006).

### Production and characterization of mAbs

**Fig. 2.**
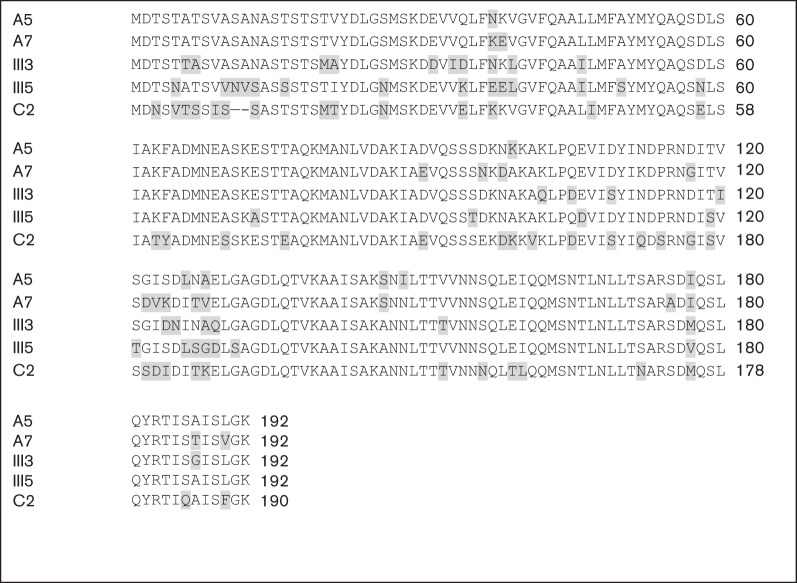
Alignment of five representative EspA amino acid sequences used for raising mAbs, using clustal 2. Source strains are A5, EspA α; A7, EspA δ; III3, EspA β; III5, EspA γ; C2, EspA ϵ. Shading indicates residues that differ from the consensus.

We chose representatives of the five major EspA proteins (Fig. 2) as targets for the production of mAbs. The initial screen of hybridoma supernatants by whole cell ELISA was designed to select only those reacting with the native target. The resulting antibodies gave various patterns of cross-reactions with the different EspA types. Three hybridomas which produced strong signals in various applications and together detected all EspA variants were selected for further work.

**Fig. 3.**
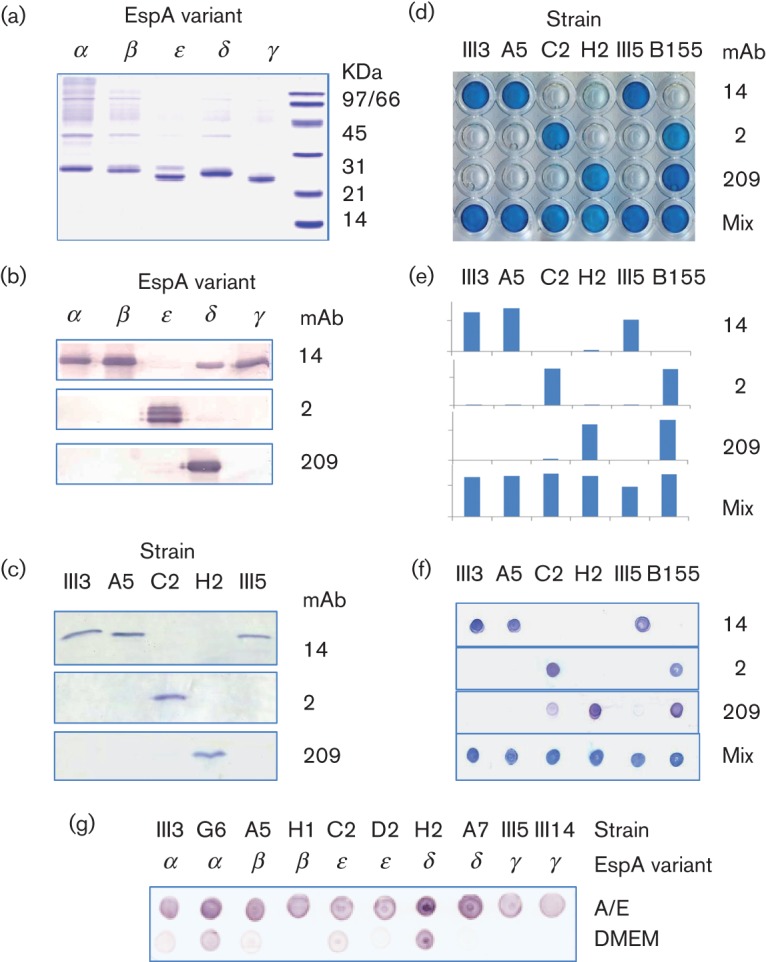
Detection of EspA variants with mAbs 14, 2 and 209. SDS-PAGE of partially purified recombinant EspA proteins stained with Coomassie blue (a). Western blots showing cross-reactivity of mAbs with recombinant EspA proteins (b) and SDS-PAGE-separated total cell protein of EPEC strains III3 (EspA β), A5 (α), C2 (ϵ), H2 (δ) and III5 (γ) (c). Direct whole cell ELISA of the same five strains plus strain B155, developed with horseradish peroxidase-antimouse antibody and TMB (d) and then developed with alkaline phosphatase-antimouse antibody and *p*-nitrophenyl phosphate; absorbance was read at 405 nm (e). Dot blots using the same strains (f). Dot blots with a mixture of three mAbs using ten strains representing the range of EspA types showing improved detection after overnight growth in A/E medium compared with DMEM (g).

Western blots against recombinant EspA confirmed that the positive ELISA signals were due to specific interactions with EspA protein (Fig. 3a, b); mAb 14 reacted strongly with EspA types α, β and γ and weakly with EspA δ, while mAb 2 and mAb 209 detected one EspA type each, ϵ and δ, respectively. Recombinant EspA ϵ produced multiple bands visible in both the Coomassie stained gel and a Western blot with mAb 2; unlike the other recombinant EspA variants, EspA ϵ contains a C-terminal histidine tag which may have resulted in translation initiation at downstream methionine codons within the *espA* gene. Western blots of cell lysates from corresponding overnight cultures of EPEC strains confirmed detection of a single band migrating at the expected position for a 21 kDa protein (Fig. 3c). The pattern of reactivity was similar to that with recombinant proteins, although no cross-reaction of mAb 14 was seen with a lysate of strain H2 carrying EspA δ.

Similar patterns of reactions were obtained with two versions of a direct whole cell ELISA of immobilized cultures, using either horseradish peroxidase-conjugated secondary antibody for rapid visual inspection of results, or alkaline phosphatase-conjugated secondary antibody for absorbance measurement using a plate reader (Fig. 3d, e). Strain B155, an A/E lesion-forming strain of unknown serotype from NICED, was included here as it was unexpectedly detected by both mAb 2 and mAb 209.

For testing large numbers of samples simultaneously during the development phase of the work, we used a dot blot. This gave results essentially identical to those obtained with ELISA (Fig. 3f), although mAb 209 also showed a slight cross-reaction in this test with strain C2 carrying EspA ϵ.

### Improved medium for EspA induction

DMEM has traditionally been used to study the virulence factors of EPEC, but we found it to be suboptimal for EspA expression in some strains in our collections. DMEM is also likely to be too expensive for routine use in developing countries. We therefore developed an alternative cheaper medium, which we called A/E (for attaching and effacing) medium, that enhanced the detection of EspA after overnight growth compared with DMEM for a representative sample of strains (Fig. 3g). In a time-course of EspA induction, most of these strains were detectable by dot blot after 4 h and all strains after 5 h in A/E medium, starting with a 1/100 dilution from an overnight culture in Luria broth (data not shown).

### Epitope mapping and IgG isotyping

**Fig. 4.**
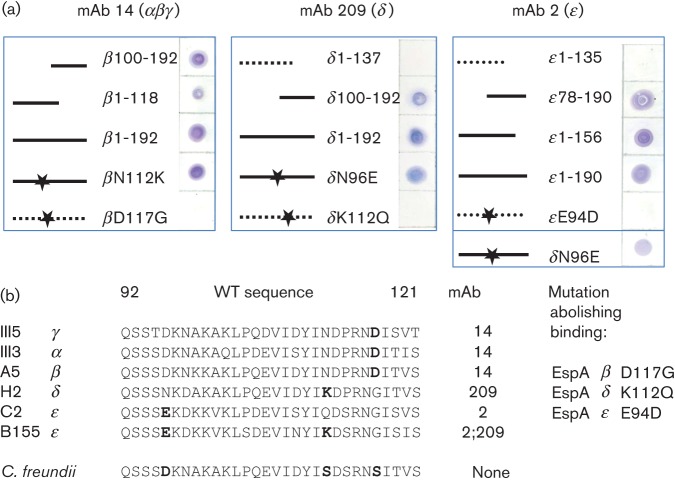
Epitope mapping. (a) Overlapping fragments of EspA β, δ and ϵ were expressed in pET28a and confirmed recombinant cultures were tested in dot blots with mAbs as indicated. Fragments not detected by antibodies are indicated by dotted lines. Mutations are marked with stars. (b) Alignment of antibody binding regions of the five major variants of EspA with the mAb that binds each variant indicated. Residues essential for binding are shown in bold type and mutations that abolished binding are shown. The EspA ϵ of strain B155 has the essential residues for binding both mAb 2 and mAb 209 and is recognized by both antibodies. The closely related EspA of *C. freundii* has none of the essential residues and is not detected by any of the three mAbs.

Overlapping fragments of *espA* from isolates A5 (β), H2 (δ) and C2 (ϵ) were tested by dot blots with mAbs 14, 209 and 2, respectively (Fig. 4a). Corresponding Western blots (not shown) confirmed that the signal was specific for EspA protein. In the case of mAb 14 the complete antigenic region was present on a short peptide between amino acids 100 and 118; the other two required larger fragments, including the central variable region of the EspA protein, for full recognition by the cognate antibody.

Individual amino acid residues were chosen for mutagenesis of the three EspA targets on the basis of sequence homology in the central region and antibody cross-reactions. The full-length mutated EspA proteins were tested in dot blots (Fig. 4a) and Western blots (not shown) as above. In all three cases mutation of a single charged amino acid within the predicted surface exposed loop of the EspA filament abolished antibody binding. Interestingly, an amino acid change in EspA δ of asparagine (N) to glutamic acid (E) at position 96, equivalent to the essential E at position 94 in EspA ϵ, resulted in recognition by mAb 2 in addition to mAb 209. Fig. 4b summarizes the results, and includes the EspA of strain B155 in the alignment, which also cross-reacted with both mAbs 2 and 209 and carries the residues essential for binding of both antibodies. Inspection of additional EspA sequences deposited in the database showed that all contained at least one of the essential amino acids required for recognition by our three mAbs. The *C. freundii* EspA sequence, on the other hand, contains none of the amino acids residues important for binding of our three mAbs. Antibody isotyping indicated IgG1 for mAb 14 and IgG2b for both mAb 2 and mAb 209.

### Sandwich ELISA and LFI platforms

**Fig. 5.**
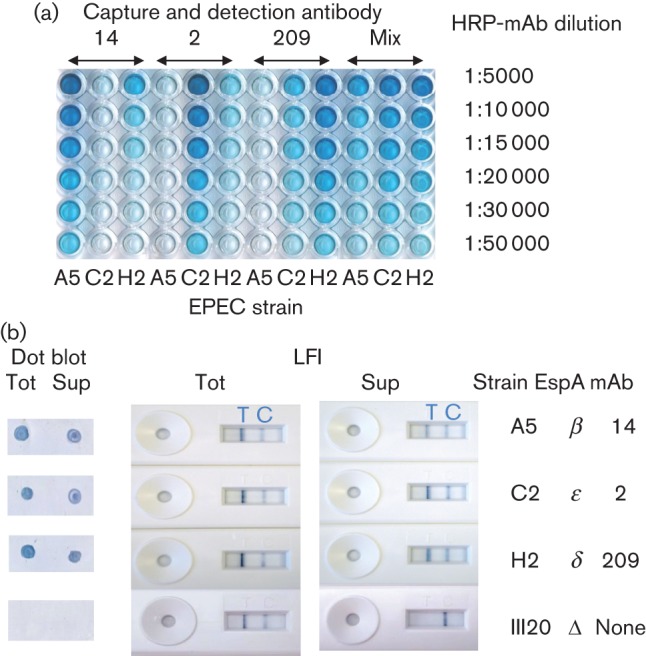
Development of sandwich ELISA and LFI for EPEC detection. (a) ELISA plates were coated with capture antibody, either individually or mixed. Representative cultures expressing EspA β (A5), EspA ϵ (C2) and δ (H2) detected by the three mAbs were grown in A/E medium overnight and added undiluted to wells in columns as shown. Horseradish peroxide-conjugated antibodies (HRP-mAb) were added at the dilutions indicated. Detection was with tetramethyl benzidine for 15 min. (b) Induced cell cultures were subjected to dot blot both before (Tot) and after (Sup) centrifugation to remove bacterial cells. Aliquots were supplemented with 0.5 M sodium chloride and loaded onto mixed antibody LFIs before (Tot) and after (Sup) centrifugation. Test (T) lines on LFIs are indicated, as are strains, EspA type and the antibody recognizing the strain. Results were recorded after 5 min.

Fig. 5a shows the results of a sandwich ELISA using both individual and mixed antibodies for capture and detection. The pattern of antibody cross-reactions with the various EPEC strains was the same as that obtained with Western and dot blots. At high antibody concentration mAb 14 (α, β, γ) showed a slight cross-reaction also with EspA δ and mAb 209 (δ) also significantly cross-reacted with EspA ϵ. The most specific antibody was mAb 2 (ϵ). The prototype ELISA developed by R-Biopharm AG uses a mixture of all three antibodies for both capture and detection.

In initial tests with the LFI devices we occasionally observed false positive bands at the test line. This is illustrated in Fig. 5b, where strain III20, an O_rough_ : H6 strain from the CBT collection (RKI no. 05-02949) that carries an *espA* deletion, gave no signal by dot blot but a positive test line by LFI, however removal of bacterial cells by brief centrifugation yielded a supernatant that was negative in both tests. By contrast, strains A5, C2 and H2, which express EspA proteins, gave positive signals in both tests with or without centrifugation. Subsequent testing of many other EPEC and EHEC strains confirmed that most, if not all, of the antigenic activity was present in the culture supernatant after overnight growth, suggesting that prior centrifugation could be used to prevent false positive results.

### Specificity and sensitivity

**Fig. 6.**
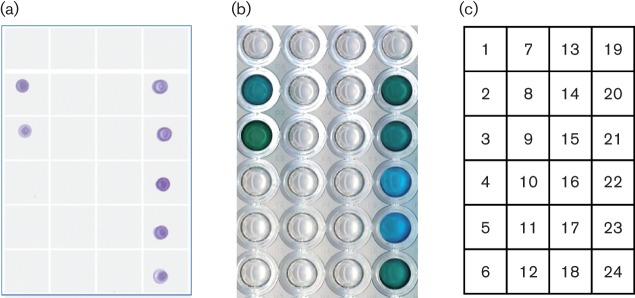
Specificity of antibodies. Cultures grown overnight in A/E medium were tested by dot blots (a) and by the prototype sandwich ELISA from R-Biopharm (b), using mixed mAbs 2, 14 and 209. The layout of micro-organisms in the tests is shown in (c): 1, EHEC O91 : H14 *eaeA*^−^; 2, EHEC O5 : H^−^; 3, EHEC O157:H7; 4, EIEC; 5, ETEC; 6, *Proteus vulgaris*; 7, *Salmonella* Typhimurium; 8, *Aeromonas hydrophila*; 9, *Morganella morganii*; 10, *Providencia stuartii*; 11, *Bacillus cereus*; 12, *A. hydrophila*; 13, *Providencia rettgeri*; 14, *Citrobacter freundii*; 15, *B. cereus*; 16, *Proteus mirabilis*; 17, *Shigella boydii*; 18 *Salmonella* Enteritidis; 19, *Pseudomonas aeruginosa*; 20, EPEC O127 : H6 (strain III3, EspA α); 21, EPEC O126 : H^−^ (strain A5, EspA β); 22, EPEC O119 : H6 (strain C2, EspA ϵ); 23, EPEC O125 : H^−^ (H2, EspA δ); 24, EPEC O157 : H7 (strain III5, EspA γ). Strains positive in both tests are indicated in bold type.

To determine the specificity of our mAbs we tested a panel of reference strains held in the collection at RKI by dot blot and sandwich ELISA following growth in A/E medium (Fig. 6a, b). While all known EPEC (*n* = 4) and EHEC (*n* = 3) strains gave positive results in both tests, all strains of the following species tested were negative: *Aeromonas hydrophila*; *Bacillus cereus*; *C. freundii*; enteroinvasive *E. coli*; enterotoxigenic *E. coli*; *Morganella morganii*; *Proteus mirabilis*; *Proteus vulgaris*; *Providencia rettgeri*; *Providencia stuartii*; *Pseudomonas aeruginosa*; *Salmonella* Enteritidis; *Salmonella* Typhimurium and *Shigella boydii*.

**Fig. 7.**
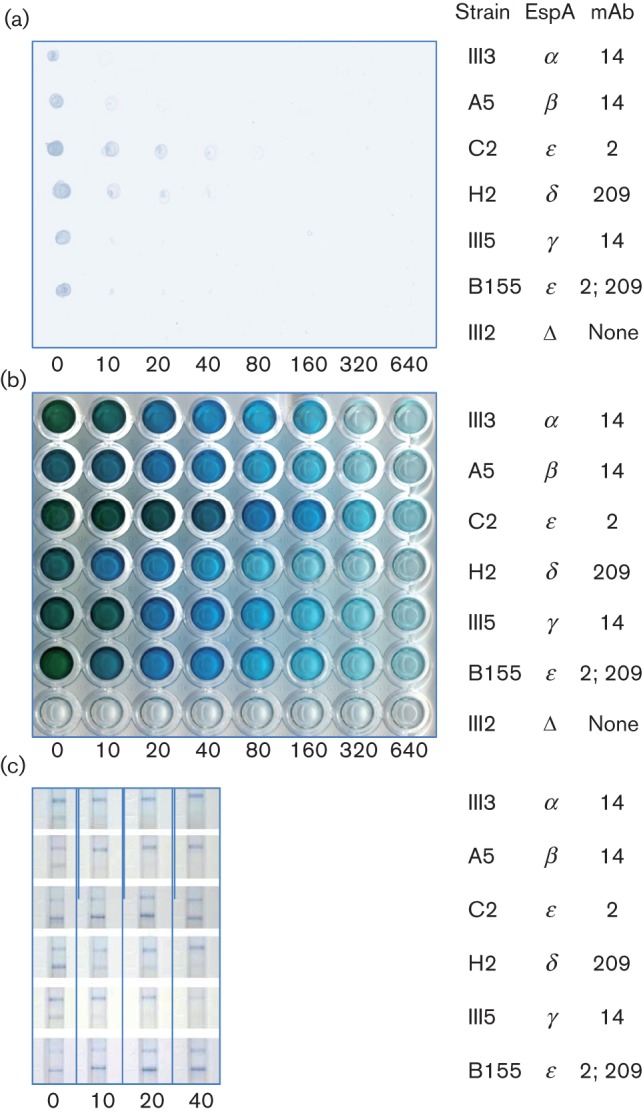
Comparison of assay sensitivities. Cultures were grown in A/E medium overnight and diluted by the factors indicated below each assay with PBS for dot blots (a) and R-Biopharm prototype ELISA (b) and with 0.5 M sodium chloride for prototype LFI using culture supernatants (c). All assays were performed with mixed antibodies; the strains tested and their cognate mAbs are indicated. Control line at the top and test line at the bottom of each panel.

To determine the relative sensitivity of the various platforms, cultures were grown overnight in A/E medium and serial dilutions in PBS were tested by dot blots and by the prototype ELISA and LFI kits (Fig. 7a–c). ELISA showed much greater sensitivity than the other two tests, with dilution of cultures to at least 160-fold giving positive signals with all strains. Strains C2 and B155, recognized by mAb 2, showed even greater detectability to a dilution of 640-fold.

### Coverage of mAbs

We tested all *eae^+^* strains in our collection for EspA detection after overnight growth in A/E medium using the three antibodies separately in triplicate dot blots. The results are summarized in Table 3. Among the 213 *eae^+^* isolates, 210 were detected with at least one antibody. Seven strains from NICED (including B155) were detected by both mAb 2 and mAb 209; DNA sequencing confirmed that their *espA* genes were identical to that of B155, encoding the variant of EspA ϵ containing both essential amino acids for recognition by the two mAbs. Of the three dot blot negative strains, one was a known EHEC strain from RKI, serotype O26 : H^−^, one was a strain from Leipzig assigned to pool 1 O-antigens associated predominantly with EHEC and the third was a strain from NICED of unknown serogroup. Amplification of *espA* DNA and sequencing of products confirmed the presence of intact coding regions for all three strains: EspA γ for the Leipzig and NICED strains and a β type for the RKI strain. Subsequent testing of these strains by the more sensitive R-Biopharm ELISA also gave negative results suggesting that EspA protein was not expressed sufficiently in these strains to be detected under these conditions.

**Table 3.** Detection of intimin-positive isolates with EspA mAbs

Serogroup	Intimin-positive strains detected by individual mAbs					
	14 (α,β,γ)	2 (ϵ)	209 (δ)	2+209 (ϵ, δ)	None	Total
O26	18	1	–	–	1 (β)	20
O55	33	1	2	–	–	36
O86	–	1	–	–	–	1
O103	8	–	5	–	–	13
O111	11	–	–	–	–	11
O113	–	1	–	–	–	1
O114	3	6	–	–	–	9
O118	1	2	–	–	–	3
O119	2	6	–	–	–	8
O125	1	–	–	–	–	1
O126	2	–	–	–	–	2
O127	5	–	1	–	–	6
O128	9	1	–	–	–	10
O142	1	–	–	–	–	1
O145	12	3	–	–	–	15
O157	7	–	5	–	–	12
Untyped	32	15	6	7	1 (γ)	61
Pool 1*	–	–	–	–	1 (γ)	1
Pool 2†	1	–	–	–	–	1
Pool 3‡	1	–	–	–	–	1
Total	147	37	19	7	3	213

Intimin-positive strains from Leipzig, RKI, Mexico and NICED were grown in A/E medium overnight and aliquots were tested in triplicate dot blots, each probed with a separate mAb. For strains not detected by an mAb, the EspA type derived from DNA sequencing of the *espA* genes is indicated in parentheses.

* O26, O103, O111, O145, O157.

† O55, O119, O125ac, O127, O128ab.

‡ O86, O114, O126, O142.

### Lack of correlation between serology and A/E virulence

The heterogeneity of EspA types within EPEC and EHEC serogroups is illustrated in Table 4. Of the 242 strains from Leipzig, isolated on the basis of serotypes classically associated with EPEC and EHEC, only 104 were shown to be *eae^+^* (intimin-positive) by PCR. The majority of these belonged to O-serogroups such as O26, O55 and O157 that are traditionally, although not exclusively, associated with EHEC rather than EPEC. Similarly, in a recent trial of the R-Biopharm ELISA kit with clinical isolates from children with diarrhoea at CMC, Vellore, only 3 out of 20 strains characterized as EPEC by serotyping were actually positive for EspA and subsequent testing of the samples by PCR confirmed the presence of *eae* in these three strains, but not in any of the other strains (Table 4). These data confirm the much-reported poor correlation between *E. coli* O-serogroup and virulence.

**Table 4.** Frequency of intimin-positive strains among O-serogroups classically associated with EPEC and EHEC

Serogroup	IMM, Leipzig		CMC, Vellore	
	Intimin-positive	Intimin-negative	Intimin-positive	Intimin-negative
O55	25	6	1	3
O128	9	22	–	–
O26	16	3	2	7
O125	1	18	–	–
O126	0	18	0	3
O145	13	4	–	–
O103	12	4	–	–
O86	1	15	–	–
O114	7	8	–	–
O127	3	10	0	1
O157	8	0	–	–
O111	0	8	0	3
O119	3	3	–	–
O118	3	0	–	–
O142	0	3	–	–
O158	0	2	–	–
Pools 1,2,3*	3	14	–	–
Total	104	138	3	17

Heterogeneity of EspA types within EPEC and EHEC serogroups in strains from IMM, Institut für Medizinische Mikrobiologie, Leipzig, Germany and CMC, Christian Medical College, Vellore, India.

* Pool 1, O26, O103, O111, O145, O157; pool 2, O55, O119, O125ac, O127, O128ab; pool 3, O86, O114, O126, O142. Intimin genes were amplified from bacterial cell extracts using universal intimin primers as described by Batchelor *et al.* (1999).

## Discussion

The EspA filament is an excellent immunodiagnostic target for infant diarrhoea caused by EPEC. It is a key virulence factor present in all EPEC strains, it can be induced to high levels in culture and its structure makes it accessible from all sides to antibodies. We have raised a panel of three monoclonal antibodies that together recognize all five currently known major variants of EspA (α, β, γ, δ and ϵ) found in EPEC and EHEC strains. Perhaps not surprisingly, one mAb detected the three most closely related EspA types α, β and γ, whereas specific antibodies were elicited by the more divergent EspA variants δ and ϵ. The three antibodies cross-reacted in Western blots with purified recombinant EspA proteins and with extracts from cultures grown in medium that induced the expression of EspA. The pattern of cross-reactions of antibodies with EspA variants was identical in ELISA using immobilized cultures. Importantly, while almost all strains known to be EPEC, on the basis of PCR of the intimin gene *eae*, expressed a protein that was recognized by one or more of our antibodies, none of a panel of non-EPEC/non-EHEC commensal and pathogenic intestinal reference strains were detected by the EspA antibodies in any test format.

EspA proved a suitable target for sandwich-type tests using the same antibody for capture and detection. In such a test only multimeric targets can be detected with a single mAb and although EspA filaments were found to be detached from the EPEC cells after overnight growth it is clear that they must remain at least partially intact to be detected in both the sandwich ELISA and LFI. The sandwich ELISA was by far the most sensitive test, even when mixtures of antibodies for capture and detection were used. Of the three mAbs used, mAb 2 gave the strongest signal for its target in all test formats, so there may be a need to adjust the proportions of the antibodies in mixed assays to optimize sensitivity. However, even before optimization, dilution of EPEC cultures up to 1/160 could be reliably detected by the sandwich ELISA. Given that an overnight culture of EPEC typically reaches a density of approximately 10^9^ cells ml^−1^, the limit of detection for the most sensitive test format is therefore 10^6^ to 10^7^ cells ml^−1^.

Results with LFIs suggest that optimization of this platform would present more of a challenge as some non-EPEC control cultures gave false positive test lines, presumably due to bacterial aggregates. This problem was solved by prior centrifugation and since the antigenic activity of EPEC cultures was present in the supernatant, a centrifugation step could safely be added to prevent false positive results without compromising the efficacy of the test. The disadvantage of introducing this additional step, however, is that it significantly reduces simplicity and convenience, which are important considerations for the adoption of the test, especially in developing countries. It may be possible to avoid this centrifugation step by using a support matrix other than nitrocellulose that does not impede the migration of bacterial cells and aggregates, but this has not yet been tested. However, the main disadvantage of LFI compared with sandwich ELISA remains its much lower sensitivity, giving positive test lines only up to ten-fold dilutions for some of the strains with the mixed antibody devices.

A crucial factor determining the sensitivity of the antibody-based tests was the growth medium used for inducing EspA expression. We noticed great variations in the expression of EspA using DMEM as the culture medium and we used some of the poor expressers as indicator strains to formulate an improved medium, A/E medium, which gave good results with many strains that had previously been undetectable or that showed very weak signals in the dot blot when grown in DMEM. Among 213 clinical isolates showing a positive PCR signal for the intimin gene *eae*, only three were not detected by our antibodies after growth in A/E medium, even with the most sensitive ELISA test. One of these was a confirmed EHEC strain and the other two belonged to O-serogroups or carried the γ variant of EspA often associated with EHEC. All three had intact *espA* coding regions, so the lack of EspA protein must have been the result of *espA* gene repression. Differences in virulence regulation between EPEC and EHEC have been reported (Mellies *et al.*, 2007). Nevertheless, we were able to detect five other confirmed EHEC strains from the RKI collection, as well as a large number of likely EHEC isolates in the Leipzig collection. Indeed, 75 of 104 intimin-positive strains from Leipzig belonged to serogroups usually associated with EHEC or shared between EPEC and EHEC.

Our screening results with clinical isolates provide further evidence for the lack of reliability of serological classification as a method of determining pathotypes of *E. coli* strains. It is clear that on the one hand a large proportion of samples are currently misdiagnosed as EPEC by serogrouping, while on the other hand an unknown number are not detected. This highlights the superiority of our immunological test for routine diagnostic purposes. Our panel of three antibodies detects all currently known variants of EspA, including minor local variants such as the unusual ϵ variant from NICED that reacted with two of our antibodies.

In summary, when used together with the improved medium for EspA induction the prototype sandwich ELISA based on a panel of three monoclonal antibodies was shown to be specific and sensitive and thus has potential as a routine diagnostic tool for the identification of EPEC. Where EHEC rather than EPEC is suspected this can be confirmed by a parallel test for shigatoxins. The level of sensitivity is sufficient for testing mixed cultures grown from stool samples of children with diarrhoea using simple visual interpretation of results without the need for a plate reader. Further work is under way in our laboratories to develop a routine protocol for the detection of EPEC in stool samples. The prototype LFI was less sensitive than the ELISA platform and so is not as promising for the identification of EPEC in mixed cultures. However, for reference and diagnostic laboratories working with pure cultures our LFI represents a valuable, informative and reliable tool in the repertoire of diagnostic tests, particularly as a replacement for conventional serology.
